# The cost of caring: emotion regulation difficulties mediate burnout and emotional overeating in nurses

**DOI:** 10.3389/fnut.2026.1831265

**Published:** 2026-05-29

**Authors:** Hanqing Zhang, Xiaoru Wu, Sheng Qiu

**Affiliations:** 1 Renmin Hospital of Wuhan University, Wuhan, Hubei, China; 2 The First Affiliated Hospital, Zhejiang University School of Medicine, Hangzhou, China; 3 Faculty of Public Health, Mahidol University, Bangkok, Thailand

**Keywords:** burnout, emotional exhaustion, depersonalization, emotion regulation difficulties, emotional overeating, nurse

## Abstract

**Background:**

Nurses are a high-risk group for occupational burnout due to chronic work-related stress, which may contribute to maladaptive eating behaviors, particularly emotional eating. However, the underlying mechanism linking burnout and emotional eating remains unclear. This study aims to examine the mediating role of difficulties in emotion regulation in the relationship between occupational burnout (emotional exhaustion and depersonalization) and emotional overeating among nurses.

**Methods:**

A cross-sectional survey was conducted among 280 nurses from a tertiary hospital between October and December 2025. The emotional exhaustion and depersonalization subscales of the Chinese version of Nurse Burnout Inventory, the 16-item Difficulties in Emotion Regulation Scale (DERS-16), and the Emotional Overeating subscale of the Adult Eating Behavior Questionnaire (AEBQ) were administered. Mediation analysis was performed using bias-corrected percentile bootstrap method with 5,000 resamples, adjusting for covariates including monthly night shifts, body mass index, years of work experience, and sex.

**Results:**

Both emotional exhaustion and depersonalization were positively associated with difficulties in emotion regulation (*β* = 0.462, *p* < 0.001; *β* = 0.268, *p* < 0.001, respectively), and difficulties in emotion regulation significantly predicted emotional overeating (*β* = 0.285, *p* < 0.001). The indirect effects of emotional exhaustion [indirect effect = 0.132, 95% BootCI (0.047, 0.224)] and depersonalization [indirect effect = 0.077, 95% BootCI (0.025, 0.141)] on emotional overeating via difficulties in emotion regulation were significant, whereas the direct effects were non-significant, indicating full mediation. The total indirect effect of both burnout dimensions through emotion regulation difficulties was 0.208 [95% BootCI (0.079, 0.339)].

**Conclusion:**

Difficulties in emotion regulation mediate the relationship between occupational burnout and emotional overeating among nurses. Burnout may increase the risk of emotional overeating by impairing nurses’ capacity for emotion regulation. Interventions targeting emotion regulation skills may help disrupt the pathway from occupational burnout to maladaptive eating behaviors.

**Implication for nursing management:**

These findings highlight the critical need for nurse managers to address emotional regulation as a key intervention target in mitigating burnout-related maladaptive eating behaviors. Nursing administrators should consider implementing regular screening programs to identify nurses with elevated burnout and emotion regulation difficulties, alongside developing targeted training workshops on emotion regulation strategies—such as cognitive reappraisal and mindfulness—as part of employee assistance programs. Furthermore, organizational efforts to reduce systemic stressors (e.g., optimizing shift schedules, improving nurse-to-patient ratios) remain essential, as they address the root causes of resource depletion. By integrating emotion regulation support into routine occupational health initiatives, nursing management can help disrupt the psychological pathway from burnout to emotional overeating, thereby promoting both the psychological well-being and long-term health of nursing staff.

## 1 Introduction

Occupational burnout is defined as a state of emotional, attitudinal, and behavioral exhaustion resulting from an individual’s inability to cope with work-related stress (1). It constitutes a significant variable in the course of professional development, and most individuals are likely to experience burnout at various stages of their career trajectories. Given high work intensity and prolonged hours, nurses are particularly susceptible to occupational burnout (2, 3). Such burnout not only affects nurses’ physical and psychological well-being but may also diminish job performance and disrupt organizational climate, thereby impacting patient safety and treatment outcomes (4). Chinese nurses face significant structural pressures that existed long before the COVID-19 pandemic. These pressures include high nurse-to-patient ratios, inadequate staffing, frequent night shifts, limited career development opportunities, and insufficient psychological support (5, 6). Such long-standing pressures have led to the continuous accumulation of physical and mental burdens among nurses, laying a deep foundation for the onset of occupational burnout. Studies have found that these persistent organizational pressures are significantly associated with increased levels of emotional exhaustion and depersonalization among nurses (7).

Since burnout is known to impair emotion regulation capacity, it is worth examining how such deficits may contribute to maladaptive coping behaviors, including emotional overeating (8, 9).

Emotion regulation broadly refers to the internal and external processes involved in monitoring, evaluating, and modifying emotional responses in service of achieving personal goals (10).

It is well established that food intake is influenced not only by physiological needs but also by emotions, particularly negative affect (11, 12). The term “emotional overeating” is commonly defined as the tendency to overeat in response to negative emotions—namely, negative emotional overeating (13).

According to frequently cited theories related to emotional overeating, such as the psychosomatic theory (14) and the affect regulation model (15), eating behavior triggered by emotions may be conceptualized as an attempt to regulate and alleviate negative emotional states in the absence of genuine physiological hunger.

“The Conservation of Resources (COR) theory (16) posits that individuals strive to acquire, maintain, and protect valued resources, and psychological stress occurs when these resources are threatened or lost. In the demanding clinical environment, nurses must continually invest emotional, cognitive, and physical resources to meet high work demands. When these investments are not adequately replenished, resources become progressively depleted. From a COR perspective, the two core dimensions of burnout—emotional exhaustion and depersonalization—can be conceptualized as an advanced state of resource loss. Emotional exhaustion reflects the depletion of emotional energy reserves, while depersonalization represents a defensive cognitive withdrawal aimed at conserving remaining resources.”

COR theory further proposes that initial resource loss triggers “loss spirals” (17), wherein depleted individuals become increasingly vulnerable to further resource erosion. Difficulties in emotion regulation can be understood as a specific manifestation of this spiral: when emotional resources are exhausted, individuals lose the internal capacity to monitor, evaluate, and modulate negative emotions effectively. In COR terms, emotion regulation constitutes a critical internal regulatory resource; its impairment signals that the loss cycle has extended from energetic depletion into the domain of self-regulatory dysfunction. Without adequate regulatory capacity, nurses are less able to buffer the emotional impact of ongoing workplace stressors, leaving them more susceptible to maladaptive coping responses (18).”

“To interrupt the loss spiral or compensate for depleted internal resources, individuals may turn to alternative means of resource acquisition—a process COR theory describes as resource substitution. Emotional overeating can be viewed as one such substitution strategy: eating palatable food provides rapid sensory pleasure and energy replenishment, temporarily alleviating negative emotional states (19). However, this strategy is inherently maladaptive because it does not address the underlying resource deficit and may create additional health burdens over time. In the present study, we therefore propose that burnout depletes emotional resources, which impairs emotion regulation capacity, which in turn drives emotional overeating as a dysfunctional attempt at resource substitution.

### 1.1 Overall hypothesis

Difficulties in emotion regulation fully mediate the relationship between nurse burnout (emotional exhaustion, depersonalization) and emotional eating. Specifically, nurse burnout does not directly trigger emotional eating but rather increases the risk of emotional eating indirectly by impairing individuals’ emotion regulation abilities.

### 1.2 Specific sub-hypotheses

H1a: Emotional exhaustion positively predicts difficulties in emotion regulation.

H1b: Depersonalization positively predicts difficulties in emotion regulation.

H2: Difficulties in emotion regulation positively predict emotional overeating.

H3a: Difficulties in emotion regulation fully mediate the relationship between emotional exhaustion and emotional overeating.

H3b: Difficulties in emotion regulation fully mediate the relationship between depersonalization and emotional overeating.

Identifying emotion regulation as a mediator may inform targeted interventions that help nurses manage burnout-related eating behavior, ultimately supporting both their psychological well-being and physical health.

## 2 Methods

### 2.1 Study design and participants

A cross-sectional descriptive design was used to survey nurses in a tertiary hospital from October to December 2025. Inclusion criteria: Registered nurses currently employed in clinical settings; ≥1 year of work experience; Voluntary participation.

Exclusion criteria: Non-clinical positions; Confirmed diagnosis of eating disorder or mental illness; Invalid responses to the questionnaire; Currently pregnant or breastfeeding. A convenience sampling method was used to recruit nurses from the tertiary hospital. The study protocol was approved by the Ethics Committee of The First Affiliated Hospital, Zhejiang University School of Medicine [Approval No. (2025B) IIT Ethics 1,117], and all participants provided informed consent.

### 2.2 Measurements

The general relevant characteristics questionnaire contains 6 items, including gender, years of work experience, height, weight, BMI, and number of night shifts.

Guided by the Conservation of Resources (COR) theory and prior empirical findings, three key psychological constructs were selected for this study: burnout (specifically emotional exhaustion and depersonalization), difficulties in emotion regulation, and emotional overeating. COR theory posits that sustained work demands deplete personal resources (16). In the nursing context, burnout represents a state of chronic resource loss, with emotional exhaustion reflecting the depletion of emotional energy and depersonalization serving as a defensive withdrawal to conserve remaining resources (1). This resource-depleted state is theorized to impair internal regulatory capacity, captured here as difficulties in emotion regulation. Finally, COR’s resource substitution tenet suggests that depleted individuals may seek alternative sources of gratification; accordingly, emotional overeating is examined as a maladaptive attempt to compensate for lost emotional resources (19).

Notably, the personal accomplishment dimension of burnout was not included because it is frequently conceptualized as a relatively independent construct (e.g., self-efficacy) that correlates only weakly with resource loss dynamics (20). Second, within the Conservation of Resources (COR) theoretical framework guiding this study, burnout is conceptualized as a state of resource depletion. EE represents the loss of emotional energy, and DP reflects defensive withdrawal to conserve remaining resources, whereas PA indicates a positive self-evaluation akin to resource gain—which is conceptually inconsistent with the resource-loss dynamic under investigation (16, 17). Similarly, emotional overeating was chosen over binge eating because it measures a continuous spectrum of affect-driven eating, making it more suitable for a general nurse population without diagnosed eating disorders (21, 22).

#### 2.2.1 Burnout

The job burnout of nurses in the cancer hospital was assessed using the Maslach Burnout Inventory-Human Services Survey (MBI-HSS). Originally developed by Leiter (23), this instrument was validated by Feng et al. (24). And has demonstrated robust psychometric properties within the Chinese nursing population. The scale comprises 22 items categorized into three dimensions: Emotional Exhaustion (EE), Depersonalization (DP), and Personal Accomplishment (PA). Each item is rated on a 7-point Likert scale, ranging from 0 (“never”) to 6 (“every day”).

Specifically, the Emotional Exhaustion dimension serves as the core component reflecting the individual stress level; Depersonalization captures the interpersonal context of burnout; and Personal Accomplishment represents the self-evaluation aspect (1). In the present study, the Cronbach’s alpha coefficient for the total scale was 0.934, while the coefficients for the three subscales ranged from 0.865 to 0.934, indicating excellent internal consistency.

“In the present study, only the Emotional Exhaustion (EE) and Depersonalization (DP) dimensions were utilized for analysis, while the Personal Accomplishment (PA) dimension was excluded. This decision was informed by established theoretical frameworks and empirical evidence. First, EE and DP are widely recognized as the ‘core’ components of burnout, representing the essence of the psychological strain and interpersonal distancing inherent in the syndrome, whereas PA is often conceptualized as a distinct psychological construct (e.g., self-efficacy) rather than a direct manifestation of burnout itself (20). Second, given this study’s focus on emotion regulation difficulties (DERS) and emotional eating (AEBQ), EE and DP—which reflect reactive emotional distress and maladaptive coping—are theoretically more proximal to the research outcomes than the more stable, evaluative dimension of personal accomplishment.” In the present sample, Cronbach’s alpha for the Emotional Exhaustion (EE) and Depersonalization (DP) subscales was 0.932 and 0.748.

#### 2.2.2 Difficulties in emotion regulation

Emotion regulation difficulties were assessed using the Chinese version of the 16-item Difficulties in Emotion Regulation Scale (DERS-16). Originally developed by Bjureberg et al. (25). The Chinese version was validated by Wang Guomeng and Liping (26). The scale consists of 16 items, with an internal consistency reliability of 0.91 and a four-week test–retest reliability of 0.90. It demonstrated significant moderate correlations with depression and anxiety scales (*r* = 0.44–0.57), indicating good criterion-related validity. In the present sample, Cronbach’s alpha for the DERS-16 was 0.925.

#### 2.2.3 Emotional overeating

“Participants’ eating traits were assessed using the Adult Eating Behavior Questionnaire (AEBQ), originally developed by Hunot et al. (22). And validated in the Chinese population by He et al. (21). The AEBQ is a comprehensive instrument designed to measure both ‘food approach’ and ‘food avoidance’ traits. It consists of 35 items across eight dimensions: Hunger, Food Responsiveness, Emotional Overeating (EOE), Enjoyment of Food, Satiety Responsiveness, Emotional Undereating (EUE), Food Fussiness, and Slowness in Eating. Cronbach’s alpha estimates of the eight subscales of the C-AEBQ ranged from 0.76 to 0.97, and the test–retest reliability coefficients of the subscales ranged from 0.50 to 0.77. Each item is rated on a 5-point Likert scale, ranging from 1 (‘strongly disagree’) to 5 (‘strongly agree’).

For the purpose of this study, we specifically focused on Emotional Overeating (EOE). We focused on EOE rather than binge eating because EOE captures a broader, non-clinical range of overeating in response to negative emotions, which is more appropriate for a general nurse population without diagnosed eating disorders. In the present sample, Cronbach’s alpha for the Emotional Overeating (EOE) subscale was 0.952.

### 2.3 Data collection

The questionnaire was distributed and collected via the online platform Questionnaire Star. The introductory page provided participants with detailed information regarding the study objectives, methodology, and instructions for completion. Participants were informed that their involvement was voluntary and anonymous, and that they could withdraw at any time without any negative consequences. To ensure data integrity, each IP address was restricted to a single submission. The survey was presented in a sequential, page-by-page format and required approximately 8–10 min to complete. In addition, attention-check items were embedded in the survey to detect inattentive responding, and responses completed in less than 3 min were flagged for review.

### 2.4 Sample size

This study employed Monte Carlo simulation for *a priori* sample size estimation. This method does not assume the distributional form of the product term ( ab ) of the indirect effect, allowing for more accurate estimation of statistical power and confidence intervals, and is the recommended approach for sample size planning in mediation analysis. Standardized path coefficients were set based on: (1) empirical evidence from similar constructs in nursing populations (27, 28); (2) and (2) Cohen’s conventional effect size benchmarks ( β=0.20 small, β=0.50 medium). Accordingly, the parameters were specified as a=0.30 , b=0.30 , and $c^{\prime }=0.10\;$ (29). With α=0.05 and a target power of 0.80, 5,000 Monte Carlo simulations conducted in R indicated that a minimum of 122 participants was required. After accounting for a 10% non-response rate, the final target sample size was set at 135.

## 3 Statistical analysis

Statistical analyses were performed using SPSS (Version 26.0) and JAMOVI (Version 2.3.28).

1. Descriptive statistics were employed to summarize the data. Frequencies and percentages were used to characterize the demographic profile of the nurses, while means and standard deviations (SD) were used to describe the scores for job burnout (Emotional Exhaustion and Depersonalization), emotion regulation difficulties (DERS), and emotional eating (EOE and EUE)
2. Pearson correlation coefficients were computed to examine the bivariate associations between job burnout, emotion regulation difficulties, and emotional overeating, providing the necessary prerequisite for mediation analysis.
3. Simple mediation analysis (equivalent to PROCESS macro-Model 4) was conducted to evaluate whether emotion regulation difficulties mediated the relationship between job burnout and emotional overeating. To test the significance of the mediation effect, the nonparametric percentile bootstrap method was applied with 5,000 resamples to estimate the 95% confidence intervals (CIs). A mediation effect was considered statistically significant if the 95% CI did not include zero.
4. All statistical tests were two-tailed, with the significance level set at 0.05.

## 4 Results

### 4.1 Participant characteristics

**Figure 1:**
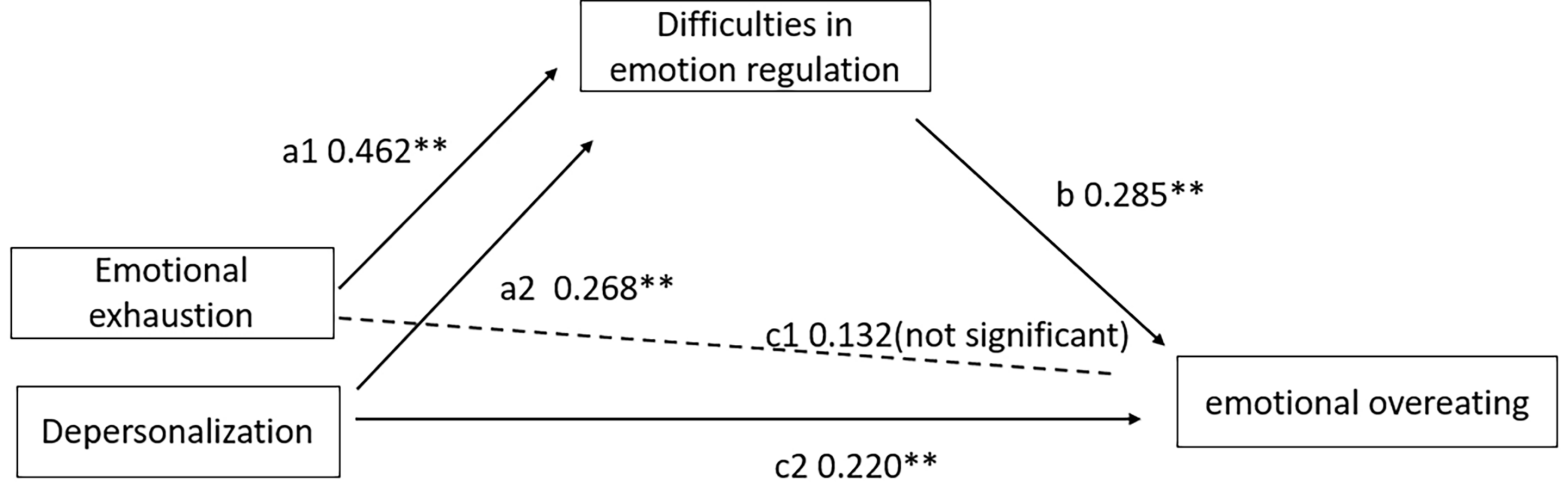
Proposed models that investigate mediated effects. ^**^p < 0.01.

A total of 280 nurses participated in this study. The sample consisted of 250 females (89.3%) and 30 males (10.7%). Regarding night shifts per month, 158 participants (56.4%) reported 0–4 night shifts, 87 (31.1%) had 5–8 night shifts, 31 (11.1%) had 9–12 night shifts, and 4 (1.4%) had more than 12 night shifts. In terms of work experience, 84 nurses (30.0%) had 1–2 years, 32 (11.4%) had 3–5 years, 54 (19.3%) had 6–10 years, and 110 (39.3%) had more than 10 years of experience. The participants worked in various departments: internal medicine (*n* = 90, 32.1%), surgery (*n* = 70, 25.0%), intensive care unit (ICU) (*n* = 69, 24.6%), and emergency department (*n* = 51, 18.2%). Table 1 summarizes the demographic and work-related characteristics of the participants (see Figure 1).

**Table 1:** General characteristics of the subjects (*n* = 280).

Frequencies of sex (*N* = 280)			
Sex	Counts	% of Total	Cumulative %
Female	250	89.3%	89.3%
Male	30	10.7%	100.0%

Frequencies of night shifts (per month) *N* = 280		
Night shifts (per month)	Counts	% of Total
1 (0–4)	158	56.43%
2 (5–8)	87	31.07%
3 (9–12)	31	11.07%
4 (>12)	4	1.43%

Frequencies of work experience (years) (*N* = 280)			
Work experience (years)	Counts	% of Total	Cumulative %
1–2	84	30.0%	30.0%
3–5	32	11.4%	41.4%
6–10	54	19.3%	60.7%
>10	110	39.3%	100.0%

Frequencies of department (*N* = 280)			
Department	Counts	% of Total	Cumulative %
ICU	69	24.6%	24.6%
Emergency	51	18.2%	42.9%
Internal medicine	90	32.1%	75.0%
Surgery	70	25.0%	100%

### 4.2 Descriptive statistics results of emotional exhaustion (EE), depersonalization (DP), Difficulties in Emotion regulation and emotional overeating

Table 2 presented the descriptive statistics results of emotional exhaustion (EE), Depersonalization (DP), Difficulties in emotion regulation and Emotional overeating.

**Table 2:** The score of emotional exhaustion; depersonalization; difficulties in emotion regulation; emotional overeating.

Descriptives	Emotional exhaustion	Depersonalization	Difficulties in emotion regulation	Emotional overeating
*N*	280	280	280	280
Missing	0	0	0	0
Mean	18.6	6.17	32.7	11.8
Median	19.0	4.00	32.5	11.0
Standard deviation	13.0	6.13	10.8	5.18
Range	47	26	50	20
Minimum	0	0	16	5
Maximum	47	26	66	25

The mean score for emotional exhaustion (EE) of burnout was 18.6 (SD = 13.0), with scores ranging from 0 to 47. Depersonalization (DP) of burnout had a mean of 6.17 (SD = 6.13), ranging from 0 to 26. Difficulties in emotion regulation showed a mean of 32.7 (SD = 10.8), with scores ranging from 16 to 66. Emotional overeating had a mean of 11.8 (SD = 5.18), ranging from 5 to 25. All variables were normally distributed based on skewness and kurtosis values (All four composite scores were approximately normally distributed, with skewness values ranging from 0.14 to 1.00 and kurtosis values ranging from −1.03 to 0.19, well within acceptable limits for parametric analysis). The Cronbach’s *α* coefficients for all scales were acceptable, indicating good internal consistency (see Table 3).

**Table 3:** Pearson correlation coefficients for study variables.

Variable	1. Emotional exhaustion	2. Depersonalization	3. Difficulties in emotion regulation	4. Emotional eating
1. Emotional exhaustion	1			
2. Depersonalization	0.664^**^	1		
3. Difficulties in emotion regulation	0.640^**^	0.578^**^	1	
4. Emotional eating	0.287^**^	0.318^**^	0.376^**^	1

^**^p < 0.01; M = Mean, SD = Standard Deviation; *N* = 280.

### 4.3 Correlation analysis revealed the interrelationships among emotional exhaustion, depersonalization, difficulties in emotion regulation, and emotional eating

This pattern of correlations satisfies the preconditions for mediation analysis (30): both burnout dimensions were significantly associated with the proposed mediator, and the mediator was significantly associated with the outcome. Notably, the correlations between burnout dimensions and emotion regulation difficulties (*r* > 0.57) were substantially stronger than those between burnout dimensions and emotional overeating (*r* < 0.32), consistent with the theoretical expectation that burnout primarily affects eating behavior indirectly through impaired regulatory capacity rather than directly. Furthermore, the slightly stronger correlation of depersonalization with emotional overeating foreshadows the differential total effects observed in the subsequent mediation analysis.

### 4.4 Mediation analysis

To evaluate the proposed mediation models, we performed ordinary least squares path analysis using the PROCESS macro (Version 4.3, Model 4). The significance of indirect effects was determined through a non-parametric percentile bootstrap method with 5,000 resamples to generate bias-corrected 95% confidence intervals (CIs) (31). Two parallel models examined the mediating role of difficulties in emotion regulation between burnout dimensions (emotional exhaustion and depersonalization) and emotional overeating, controlling for night shifts, BMI, years of work experience, and sex. Continuous variables were standardized; standardized coefficients (*β*) are reported, with full details in Table 4.

**Table 4:** Mediating role of difficulties in emotion regulation in the relationships of emotional exhaustion and depersonalization with emotional eating (*N* = 280).

Path	Effect	*B*	SE	*β*	95% CI	*p*
Emotional exhaustion → difficulties in emotion regulation	*a*	0.462	0.060	0.462	0.343, 0.581	<0.001
Depersonalization → difficulties in emotion regulation	*a*	0.268	0.060	0.268	0.150, 0.386	<0.001
Difficulties in emotion regulation → emotional eating	*b*	0.285	0.075	0.285	0.137, 0.434	<0.001
Emotional exhaustion → emotional eating						
Indirect effect (via difficulties in emotion regulation)	*a*·*b*	0.132	0.045^†^	0.132	0.047, 0.224^*^	0.003
Direct effect	*c*′	~0	0.083	~0	−0.163, 0.163	0.999
Total effect	*c*	0.132	0.077	0.132	−0.020, 0.284	0.089
Depersonalization → emotional eating						
Indirect effect (via difficulties in emotion regulation)	*a*·*b*	0.077	0.030^†^	0.077	0.025, 0.141*	0.011
Direct effect	*c*′	0.144	0.077	0.144	−0.009, 0.296	0.064
Total effect	*c*	0.220	0.077	0.220	0.070, 0.371	0.004

All models adjusted for night shifts per month, BMI, years of work, and sex. Bootstrap confidence intervals (CI) are based on 5,000 resamples and are bias-corrected.

^†^BootSE (standard error of the indirect effect).

Bootstrap CI for the indirect effect.

^*^p < 0.05.

Emotional exhaustion was a significant positive predictor of difficulties in emotion regulation (*β* = 0.462, *p* < 0.001). These difficulties, in turn, significantly predicted increased emotional overeating (*β* = 0.285, *p* < 0.001). The total effect of emotional exhaustion on emotional overeating was not statistically significant (*β* = 0.132, *p* = 0.089). However, the bootstrap analysis revealed a significant indirect effect through difficulties in emotion regulation [indirect effect = 0.132, 95% BootCI (0.047, 0.224)]. Following the statistical frameworks. A significant indirect effect confirms mediation regardless of the total effect’s significance. As the direct effect was non-significant (*β* ≈ 0, *p* = 0.999), the relationship between emotional exhaustion and emotional overeating is fully mediated by emotion regulation difficulties (31, 32).

Depersonalization significantly predicted difficulties in emotion regulation (*β* = 0.268, *p* < 0.001). The total effect of depersonalization on emotional overeating was also statistically significant (*β* = 0.220, *p* = 0.004). A significant indirect effect via difficulties in emotion regulation was observed [indirect effect = 0.077, 95% BootCI (0.025, 0.141)]. Upon including the mediator in the model, the direct effect of depersonalization on emotional overeating became statistically non-significant (*β* = 0.144, *p* = 0.064). Consequently, these results support a complete mediation model for the depersonalization pathway within this sample, indicating that its influence on overeating is fully channeled through the mediator.

## 5 Discussion

The results of the mediation analysis demonstrated that difficulties in emotion regulation consistently served as a full mediator in the relationships between both dimensions of burnout (emotional exhaustion and depersonalization) and emotional overeating. These findings support our primary hypothesis that the impact of occupational burnout on maladaptive eating behaviors is not direct but is fully channeled through the impairment of internal emotion regulation capacities.

After controlling for confounding factors including the number of night shifts per month, BMI, years of work, and sex, the results partially supported the mediation hypothesis, revealing the mediating role of difficulties in emotion regulation in the relationships between emotional exhaustion and emotional overeating, as well as between depersonalization and emotional overeating.

Specifically, the full mediation effect observed for emotional exhaustion underscores the role of resource depletion. Within the framework of Conservation of Resources (COR) theory, emotional exhaustion represents the terminal stage of emotional energy drain. When nurses reach this state of depletion, they lose the critical psychological resources necessary to monitor and modulate their emotions effectively. Consequently, the heightened risk of emotional overeating arises not from the exhaustion itself, but from this subsequent collapse of self-regulatory strength, leading individuals to use food as a primary tool for affect regulation.

For depersonalization, the findings also supported a complete mediation pathway via emotion regulation difficulties. From a COR perspective, depersonalization acts as a defensive cognitive withdrawal aimed at conserving remaining emotional resources. However, this psychological distancing creates a state of “emotional numbness” or detachment, which paradoxically hinders the individual’s ability to accurately identify and manage internal emotional cues. Our results indicate that this impairment in regulatory capacity is what ultimately drives the transition from professional detachment to maladaptive eating as a form of resource substitution.

Meanwhile, we found that the scores of the two dimensions of emotional exhaustion and depersonalization in the study of nurses’ occupational burnout were slightly higher than those in other studies. This also confirms the view in previous correlation studies that burnout symptoms are significantly associated with binge eating tendencies (33), and we found that difficulty in emotion regulation is a mediator between occupational burnout and emotional overeating. This means that job burnout does not directly cause people to overeat; rather, it impairs people’s ability to manage negative emotions, forcing them to cope by eating. This may provide insights for future interventions to improve the eating behaviors of individuals with high levels of job burnout.

It is important to clarify that while our study utilizes COR theory as a conceptual framework, we did not directly quantify psychological or energetic resources. Instead, difficulties in emotion regulation were examined as a manifestation of impaired internal regulatory capacity resulting from burnout. Our findings align with COR theory’s propositions regarding resource depletion and substitution, although the specific ‘loss spiral’ mechanisms remain theoretically inferred rather than empirically tested in this cross-sectional design. Future research using longitudinal designs and direct resource measures would be valuable to further validate these theoretical claims.

## 6 Limitation

First, limitations of cross-sectional design. This study employed a cross-sectional survey, which precludes inferring causal temporal relationships between variables. Although mediation analysis tested indirect effects based on theoretical assumptions, bidirectional associations may exist between emotional exhaustion, difficulty regulating emotions, and emotional overeating.

Second, the study did not examine the direction of dietary behavior differentiation. While focusing solely on emotional overeating, prior research indicates that stress can also lead to reduced appetite (19, 34). Future studies should incorporate “emotional undereating” as an outcome variable to investigate whether emotional regulation difficulties mediate differently in occupational burnout and bidirectional dietary changes. This would contribute to constructing a more comprehensive theoretical model linking occupational burnout and dietary behavior.

Third, this study relied exclusively on self-report measures, which may introduce social desirability bias or recall bias. Nurses might underreport their levels of burnout or emotional overeating due to professional stigma or perceived social expectations. Future research should consider incorporating objective physiological markers or behavioral observations to supplement self-reported data and enhance the robustness of the findings.

Fourth, the study was conducted in a single tertiary hospital, which may limit the generalizability of the findings to nursing staff in different hospital tiers (e.g., primary or secondary hospitals) or diverse healthcare systems.

Fifth, although sex was adjusted for as a covariate in our models, the sample was predominantly female (89.3%). We did not examine whether sex moderates the relationships between burnout, emotion regulation, and eating behavior. Future multi-site studies with more balanced gender distributions are needed to test these potential moderating effects and enhance external validity.

## 7 Conclusion

In conclusion, this study provides evidence that difficulties in emotion regulation serve as a key mechanism linking occupational burnout to emotional overeating among clinical nurses. Within the current sample and statistical framework, the results suggest that both emotional exhaustion and depersonalization influence eating behavior indirectly through the erosion of emotion regulation capacities. While these findings point toward a full mediation process, they highlight that interventions should prioritize the enhancement of nurses’ emotion regulation skills alongside organizational efforts to reduce burnout.

At the practical level, these findings provide clear directions for intervention in eating problems among populations with high burnout: in addition to alleviating burnout symptoms themselves, interventions should more directly target the enhancement of emotion regulation capacity. It is recommended to implement interventions such as emotion awareness training and cognitive reappraisal strategy instruction among nurse populations to interrupt the pathway from burnout to problematic eating behaviors.

## Data Availability

The datasets presented in this study can be requested from the corresponding author upon reasonable request.
